# Nephele: genotyping via complete composition vectors and MapReduce

**DOI:** 10.1186/1751-0473-6-13

**Published:** 2011-08-18

**Authors:** Marc E Colosimo, Matthew W Peterson, Scott Mardis, Lynette Hirschman

**Affiliations:** 1 The MITRE Corporation, 202 Burlington Rd, Bedford MA 01730, USA

## Abstract

**Background:**

Current sequencing technology makes it practical to sequence many samples of a given organism, raising new challenges for the processing and interpretation of large genomics data sets with associated metadata. Traditional computational phylogenetic methods are ideal for studying the evolution of gene/protein families and using those to infer the evolution of an organism, but are less than ideal for the study of the whole organism mainly due to the presence of insertions/deletions/rearrangements. These methods provide the researcher with the ability to group a set of samples into distinct genotypic groups based on sequence similarity, which can then be associated with metadata, such as host information, pathogenicity, and time or location of occurrence. Genotyping is critical to understanding, at a genomic level, the origin and spread of infectious diseases. Increasingly, genotyping is coming into use for disease surveillance activities, as well as for microbial forensics. The classic genotyping approach has been based on phylogenetic analysis, starting with a multiple sequence alignment. Genotypes are then established by expert examination of phylogenetic trees. However, these traditional single-processor methods are suboptimal for rapidly growing sequence datasets being generated by next-generation DNA sequencing machines, because they increase in computational complexity quickly with the number of sequences.

**Results:**

Nephele is a suite of tools that uses the complete composition vector algorithm to represent each sequence in the dataset as a vector derived from its constituent k-mers by passing the need for multiple sequence alignment, and affinity propagation clustering to group the sequences into genotypes based on a distance measure over the vectors. Our methods produce results that correlate well with expert-defined clades or genotypes, at a fraction of the computational cost of traditional phylogenetic methods run on traditional hardware. Nephele can use the open-source Hadoop implementation of MapReduce to parallelize execution using multiple compute nodes. We were able to generate a neighbour-joined tree of over 10,000 16S samples in less than 2 hours.

**Conclusions:**

We conclude that using Nephele can substantially decrease the processing time required for generating genotype trees of tens to hundreds of organisms at genome scale sequence coverage.

## Background

In the post-genomic era, as sequencing becomes ever cheaper and more routine, biological sequence analysis has provided many useful tools for the study and combat of infectious disease. These tools, which can include both experimental and computational methods, are important for molecular epidemiological studies [1-3], vaccine development [4-6], and microbial forensics [7-9]. One such method is genotyping, the grouping of samples based on their genetic sequence. This can be done experimentally [10-12] or computationally, either by identifying genetic signatures (nucleotide substrings which are only found in a single group of sequences) [13], or on the basis of genetic distance among the sequences [14-16]. These methods allow a researcher to split a group of sequences into distinct partitions for further analysis. In a forensics context, genotyping a sequence can yield clues on where the sequence comes from. In surveillance, genotyping can be used to examine the evolutionary footprint of a pathogen, for example, to identify areas where certain vaccines and other countermeasures should be used.

Sequence-based comparison involves three major steps. The first is to choose a set of sequences to study, based on some criteria, such as strain, time period or geographic region. Ideally, this set can be easily extracted from a well-populated reference database, containing not only the sequence data for the samples of interest, such as a particular serotype of *Influenza*, but also sufficient metadata. For infectious diseases, types of metadata include geospatial and temporal co-ordinates, host information, and pathogenicity. Once the appropriate dataset is chosen, the samples are compared and clustered in sequence space. From here, the metadata associated with the sequences is used to assess the evolutionary landscape of the organism or pathogen [17].

### Sequence Comparison Methods

Traditionally, the first step in performing sequence comparisons is to generate a multiple sequence alignment (MSA) from the sequences of interest. This is most often done using heuristics found in utilities such as CLUSTAL W [18], MUSCLE [19], T-COFFEE [20], and ProbCons [21]. The dynamic programming solution, which can find the mathematically but not necessarily biologically correct solution, quickly becomes impractical with the sample sizes used in any meaningful analysis. A recent review [22] examined many of the issues in producing these alignments, most notably the trade-offs between alignment accuracy, time, and computational expense. Many of the most accurate algorithms cannot be used on a large number of sequences, or on very lengthy sequences, and were only recommended for sets of less than 100 sequences.

Because the alignment is dependent on each of the sequences from which it is calculated, the alignment must be recomputed whenever a new sequence is added. This becomes problematic for surveillance applications, where new sequences will be added constantly. While this problem has been mitigated to some extent using with algorithms such as Near-Alignment Space Termination (NAST) [23], this still adds a level of complexity if the dataset is continually growing, as is the case with Influenza and other infectious diseases. Another issue is that different heuristics will yield different alignments -- they are only designed to find an acceptable answer, not the optimal alignment. While methods have been developed to find a "consensus alignment" [24] from a set of alignments, this requires a good deal of time and computing power.

The composition vector (CV) [25] method has been used to describe DNA/RNA and protein sequences as vectors, using the distance between these vectors as the genetic distance. This method involves using a sliding window to represent each sequence as a vector, where each element of the vector is calculated based on the actual and expected frequency of the k-mer (DNA/protein subsequence of length k) observed in that window. The vector representation allows the distance between two sequences to be calculated with any standard distance metric. The CV method was shown to produce trees which matched established taxonomies, as inferred from the 16S RNA segment by more conventional alignment-based methods [26]. The CV method was later expanded into the complete composition vector (CCV) method [27], which uses sliding windows over a range of lengths to describe the sequence. Since these methods do not require alignments to be calculated, distances calculated between sequences remain constant, rather than being dependent on the set of sequences being examined, making these methods ideal for the handling of rapidly growing datasets. No molecular models need be used to calculate distances -- distances are calculated using any distance metric that can be used to calculate the distance between vectors.

The next step in sequence analysis is the clustering of the sequences. Traditionally, this is done by inferring a phylogenetic tree. Tools for this purpose include PHYLIP [28], PAUP* [29], or POY [30]. This work was initially performed using distance-based methods, such as the UPGMA or neighbour-joining algorithms [31], or cladistic methods such as Maximum Parsimony. As computational power increased, methods that inferred trees based on models of evolution were used. These include the Maximum Likelihood technique, as well as Bayesian Inference. While these methods produce phylogenetic trees, which provide a useful visualization, any further analysis and grouping must be performed manually. As the number of sequences to compare increases, this becomes more and more difficult. In fact, there has been much recent research into new methods to visualize phylogenetic trees with large numbers of leaves [32,33]. In addition, the phylogenetic tree view proves difficult to integrate with the metadata. For example, a recent paper discussing the spread of H5N1 Avian Influenza used Google Earth to draw a phylogenetic tree on top of the globe [34]. While this visualization works well for a small number of samples, it is ineffective for larger datasets, due to the "busyness" of the visualization.

### Computational Genotyping

An alternative to the pure phylogenetic approach is computational genotyping. This involves partitioning the set of sequences into discrete groups, based on some criteria. This can be based on differences between known subtypes, such as tandem repeats or single nucleotide polymorphisms, or by genetic distance. In the case of genotyping based on distance, this becomes a clustering problem. In 2007, Frey and Dueck published a paper on a new clustering algorithm known as affinity propagation clustering [35]. In contrast to other clustering algorithms, such as k-means and Expectation Maximization (EM), the affinity propagation algorithm does not require the user to explicitly select a given number of exemplars at the start of clustering. Instead, affinity propagation simultaneously considers all points as potential exemplars, using an initial preference to determine the sensitivity, and therefore the number of clusters. This eliminates the need for large numbers of runs to determine the ideal number of clusters and any dependence on initial conditions seen in other partition clustering algorithms. Furthermore, this algorithm allows the user to set the preference for each data point. This is useful for a scenario where a partial set of representative samples are known, but there may be other exemplars along with these in a data set. The affinity propagation has been tested on geospatial, text, and gene expression data and showed improvements in both speed and accuracy over other clustering algorithms.

The main advantage of an automated computational genotyping method is that it gives the researcher the ability to combine a measure of sequence similarity (cluster membership) with the metadata. It is this metadata that yields the most information about a sample. A phylogenetic tree will tell what samples are close in sequence space, but any further inference is made using the metadata. By separating the sequences into discrete groups, the researcher is given much more flexibility to visualize the data and associated metadata.

### MapReduce

MapReduce [36] is the software framework developed by Google™ to support parallel distributed execution of their data intensive applications. MapReduce is designed for fault-tolerant computations with extremely large datasets. MapReduce is divided into two major phases called map and reduce, separated by an internal shuffle phase of the intermediate results. Hadoop is an open-source version of MapReduce implemented in Java and sponsored by Amazon™, Yahoo™, and other major vendors. Recently, MapReduce has been used for sequence and phylogenetic applications. For example, CloudBurst uses Hadoop for parallel short read-mapping for use in a variety of biological analyses including SNP discovery, genotyping, and personal genomics [37]. The Genome Analysis Toolkit uses the MapReduce paradigm for shared memory platforms [38]. MrsRF (MapReduce Speeds up RF) is a multi-core, multi-machine algorithm that generates t × t Robinson-Foulds distance matrix between t trees [39] using Phoenix [40], a MapReduce implementation for shared memory multi-core platform, and OpenMPI [41]. These uses indicate that MapReduce is a promising tool to help solve the computational challenges with large datasets.

### Nephele

In this paper, we describe a scalable complete genotyping system that brings together the complete composition vector and affinity propagation algorithms to produce genotypes from *Influenza *A sequences. The system has been tested on a variety of *Influenza *A and *Actinomycetes *genome data. In addition to providing discrete clusters representing genotypes, we use methods that produce trees that closely match the topologies of trees inferred using traditional phylogenetic methods, in order to provide scientists with a more familiar visualization.

## Implementation

### Datasets

The *Influenza *dataset used to develop our methods was that of Holmes *et al*. [42]. The clades and reassortment events found in these samples were discussed in detail, providing eight sets of sequences (one for each gene studied in the paper) for verification of our methods. This dataset consists of 155 samples, taken from New York State during the 1999-2000, 2001-2002, 2002-2003, and 2003-2004 flu seasons. The complete coding sequences are available, as well as the date and county of collection for these strains. We also used HA segments from H1N1 (1141) and H3N2 (2201) parsed from GenBank's viral division (gbvrl). For testing our implementation, we used 10,270 16S samples from GreenGenes (core_set_aligned.fasta retired on 07 February 2007; http://greengenes.lbl.gov/).

To test our methods, two additional datasets were identified. A set of 94 sequences representing WHO expert-defined genotypes (http://www.who.int/csr/disease/avian_influenza/guidelines/nomenclature/) was used to validate our methods, and another dataset representing an 2007 *Influenza *outbreak in Europe was chosen to demonstrate the utility of the computational genotyping approach for microbial forensic analysis (Additional File 1).

We also used 27 full length genomes of Actinomycetes bacteria from the Broad Institute along with their computed concatenated protein sequences, downloaded from the Tuberculosis Database (TBDB) [43].

### Complete Composition Vector

The method used is based on that of Wu et al. [44]. Each sequence, S, of a given length L, can be broken into L -- k + 1 overlapping substrings of length k. For each substring α, the probability of occurrence is calculated as

$$p\left(\alpha \right)=\frac{f\left(\alpha \right)}{L-k+1},$$

where f(α) is the frequency of substring α in S. Next, the expected probability, q is calculated using a Markov model described by Brendel, Beckmann, and Trifonov, which takes into account the probabilities of length-(k-1) and length(k-2) strings [45].

$$q\left(\alpha \right)=\frac{p\left(\alpha_{1}\alpha_{2}\ldots \alpha_{k-1}\right)p\left(\alpha_{2}\alpha_{3}\ldots \alpha_{k}\right)}{p\left(\alpha_{2}\alpha_{3}\ldots \alpha_{k-1}\right)}$$

This is designed to highlight the role of selective mutation, and it was found that phylogenetic trees produced without subtracting the background via the Markov model were not consistent with traditional approaches [25].

The composition value, π, for substring α is defined as:

$$\pi \left(\alpha \right)=\left\{\begin{array}{c} p\left(\alpha \right)∕q\left(\alpha \right)-1\\ 0 \end{array}\;\right)\begin{array}{c} q\neq 0\\ q=0. \end{array}$$

The k^th ^composition vector, V_k_(S), is comprised of the composition values for all possible substrings of length k. For amino acid sequences, V is of length 20 k, and for DNA/RNA, V is of length 4 k. This method has been shown to produce trees which match known taxonomies [26].

In 2004, Wu et al. extended the CV approach into the complete composition vector (CCV) [27]. This method combines the composition vector approach with the idea of the complete information set, in order to supplement any information loss from the background subtraction in the CV method [46]. The CCV is defined as the sequence of composition vectors from 3 to M, where M is a pre-determined constant.

For all experiments described in this paper, the complete composition vectors were calculated with M = 9. In addition, the revised relative entropy string selection string scoring scheme described by Wu *et al*. [44] was employed to reduce the dimensionality of the vectors. This is calculated as

$$RE\left(\alpha \right)=\sum_{i}^{n}\left|\pi \left(a,i\right)\right|ln\left|\frac{\pi \left(\alpha ,i\right)}{\Pi \left(\alpha \right)}\right|,$$

where Π represents the complete composition vector calculated from the concatenation of all n sequences in the dataset. In summary, this method evaluates the information content associated with each possible substring, and the most informative substrings are chosen for inclusion in the analysis. The number of n-mers used for distance calculations was chosen based on the dataset: if the absolute revised relative entropy was below 1.0, the substring was not used for any further calculations.

Once the final set of n-mers is chosen, the vectors are normalized by calculating the Z-score for each n-mer. From these normalized vectors, the distance matrix is then calculated. For each pair of samples, the distance between the normalized complete composition vectors Vi and Vj is calculated using cosine distance:

$$Dij=\frac{\frac{v_{i}v_{j}}{\left|v_{i}\right|\left|v_{j}\right|}+1}{2},$$

**Figure 1:**
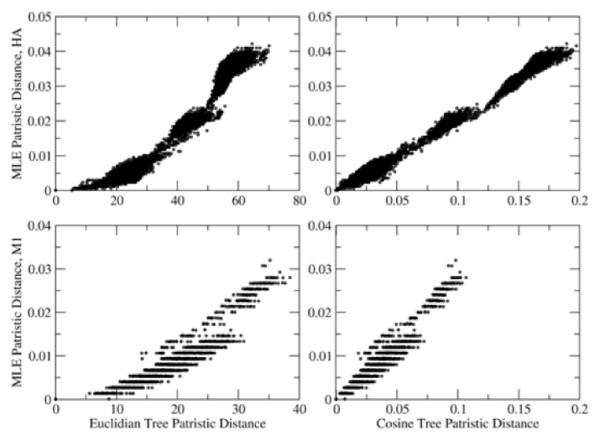
**Comparison of CCV and Maximum Likelihood Trees**. Patristic Distance plots of trees produced by Maximum Likelihood (Y axes) vs. those produced by CCV and cosine/Euclidian distance measures. (X axes).

We also experimented with using the Euclidian distance, calculated as

$${D_{i}}_{j}=\sqrt{\sum_{k=1}^{n}{\left(V_{i}\left(k\right)-V_{j}\left(k\right)\right)}^{2}},$$

where n is the number of substrings kept after the substring selection (Figure 1). The CCV and distance calculation code was written in Java (1.5+), using custom classes to save space and memory. Experiments were run on Apple dual quad-core Intel Mac Pro with 8 GB running OS × 10.6. Additional testing was done under CentOS 5.5 and Ubuntu 9.4 Linux distributions.

### Affinity Propagation Clustering

The input to the affinity propagation clustering algorithm is a similarity matrix. For Euclidian and Manhattan distances, the similarity is represented by the negative of the distance, while for cosine distances, the similarity is found by subtracting the distance matrix from 1. To determine the optimal preference, the mean silhouette value was used. This value is a measure of how similar a given sample is to others in the same clusters, versus samples found in other clusters. It ranges from 1 (sample is well-clustered) to -1 (the sample is found in an incorrect cluster) and is calculated as:

$$s\left(i\right)=\frac{b_{i}-a_{i}}{\text{max}\left(a_{i},b_{i}\right)},$$

Where a_i _is the sample's average distance to the other samples in its cluster and b_i _is the minimum average distance between the sample and the samples in each of the other clusters. The developers of the algorithm recommend using the minimum similarity between samples for a low number of clusters, and the median similarity for a moderate number of clusters. The preference resulting in the optimal partitioning, using the average silhouette value as a measurement was chosen from a set of four preferences spanning the minimum and median similarity. Affinity propagation was performed using the MATLAB function available at the authors' website (http://www.psi.toronto.edu/affinitypropagation/), with the default parameters or with our re-implementation written in Java as part of Nephele.

### Parallelization with Hadoop

We also implemented most of our CCV code for execution as a series of nine MapReduce jobs using the open-source MapReduce implementation Hadoop (http://hadoop.apache.org/). MapReduce is not ideal for the generation of neighbour-joined trees or affinity propagation. The MapReduce paradigm depends on the maps not dependent on any other data than what they are given. Both the neighbour-joined trees and affinity propagation algorithms depend on shared states, which breaks the MapReduce paradigm. However, Nephele provides a Message Passing Interface (MPI) version of Panjo, a neighbour-joining algorithm, that is able to handle very large trees [47]. Our version accepts row packed matrices instead of column packed, because it was easier to generate them as opposed to column packed matrices using MapReduce. All experiments were run on a Rocks (http://www.rocksclusters.org/) cluster running CentOS 5.4 on Intel Core 2 Quad and Core 2 Duo processors with 8 and 4 GB of memory, respectively, using Java 1.5 and Hadoop 0.20.1.

## Results and Discussion

### Genotyping the New York Dataset: Results, Computation Time and Choice of Distance Metric

The dataset used by Holmes and colleagues to study reassortment events throughout New York State provided a dataset to use to build and refine the genotyping methods. This set included 155 full genomes of H3N2 found in New York state between 1999 and 2004, collected as part of the *Influenza *Genome Sequencing Initiative [48], a worldwide sequencing initiative (this project has also sampled from the southern hemisphere, in Australia and New Zealand). Since the complete genome for each of the samples in this set was sequenced, this provided genes, with differing rates of evolution to test the pipeline.

Clustering was performed on the eight genes studied in detail in the paper (HA, M1, NA, NP, NS1, PA, PB1, PB2) as described in the Implementation section. Cluster counts ranged from 5 (NP, PB2) to 15 (PB1), with clusters ranging in size from 1 (representing an outlier in the dataset) to 36. The results from the affinity propagation clustering matched the clade structure of the trees. The trees for all eight genes, colored by cluster membership, can be seen in Additional File 2. All phylogenetic trees in this work were produced using TreeViewJ [49]. The segmented nature of the *Influenza *genome adds a level of complexity to the genotyping problem. For each sample, the set of clusters for the eight genes can be used to define a cluster "profile," which represents the genotype defined by the complete genome for that sample. This profile represents the composite genotype for that sample. For the 155 samples in the dataset, 32 genotypes were identified. From these, the groups of samples identified by Holmes and colleagues to be involved in reassortment events were found as distinct genotypes.

One of the major advantages of the complete composition vector approach over traditional phylogenetic methods is the speed of analysis. For the individual gene segments in the test dataset (155 sequences), execution times ranged from 1.50 minutes (M1) to 2.25 minutes (NA) In contrast, alignment times using MUSCLE were on the order of 5-10 minutes, and inference of maximum likelihood trees took roughly 20 minutes per gene If the genes are concatenated together to create a full genome sequence, the gains are even more impressive -- trees were produced in a few minutes with the CCV-based approach, rather than hours for traditional alignment-based methods. This is consistent with initial results from composition vector based approaches, which focused on inferring trees for complete prokaryotic genomes [26].

**Figure 2:**
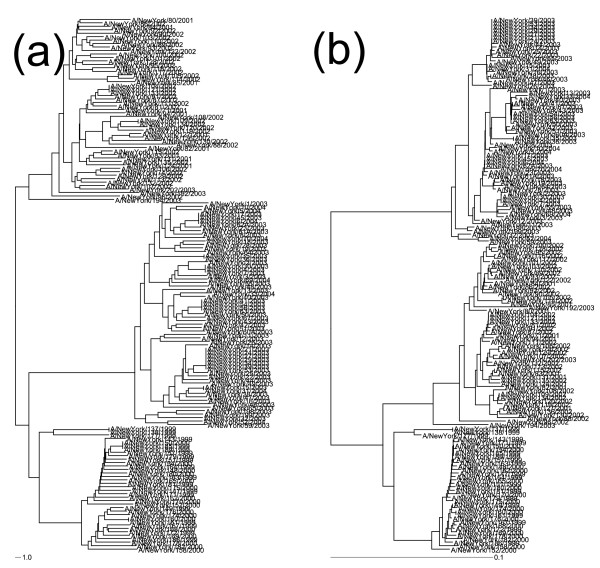
**Comparison of Distance Metrics Used to Create Trees**. Phylogenetic trees of the HA gene from the New York State dataset constructed with (a) Euclidian and (b) cosine distances. Note the long leaf-leaf distances on tree (a).

In order to determine the ideal distance metric to use for clustering, the patristic distances (distances between leaves along the branches) of the phylogenetic trees inferred using the neighbour-joining algorithm on distances calculated using cosine and Euclidian distances were compared (see Implementation section for details of computation). Patristic distances (the distance between two leaves along the branches of a tree) were calculated using the TreeDistanceMatrix methods from the Phylogenetic Analysis Java Library [50]. Patristic distances for each gene and distance measure were plotted against each other. Figure 1 shows the patristic distance (distance between leaves along a tree) plots for HA and M1, which represent rapidly mutating and slowly mutating genes, respectively. It is clear that trees produced using the neighbour-joining algorithm on cosine distance matrices produce trees that are the most similar to the Maximum Likelihood trees, while the Euclidian distance metric produces trees which have overly large distances near the leaves of the tree, as shown in Figure 2. The cosine distance is shown to produce trees whose patristic distance has a linear relationship with that of the tree produced by maximum likelihood, while the trees produced using Euclidean distance show a higher-order relationship. These indicate that while the Euclidian distance has been used as a distance metric for the majority of previously published work involving composition/complete composition vectors [15,44], it appears that cosine distance provides a better correlation with trees produced by traditional phylogenetic methods.

### Clustering on H5N1 Standard Nomenclature Dataset for Validation

In 2001, the World Health Organization (WHO), along with the World Organization for Animal Health (OIE) and Food and Agriculture Organization of the United Nations (FAO) released, in poster form, a standard nomenclature system for the various lineages of *Influenza *H5N1 found in over 50 countries throughout the world This nomenclature is intended to replace the current nomenclature used in publications, where samples are often identified by the location of the earliest sample with the closest genetic similarity (for example, "Fujian-like" or "Quinghai lineage"). Alignments of 904 HA sequences were created, and clades were chosen from the tree based on a set of rules. These clades, developed to define a new standard nomenclature, provided an opportunity to blind test set our genotyping system.

**Figure 3:**
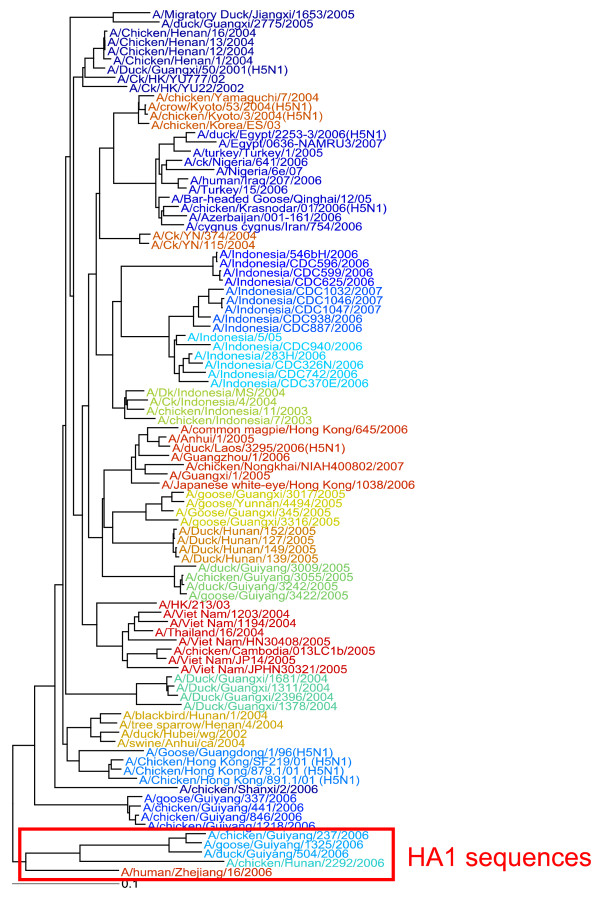
**Clustering of Influenza Dataset**. Phylogenetic tree of the WHO Dataset, colored by cluster membership. The shorter HA1 sequences are boxed in red.

In addition to the complete 904 sample dataset, the authors provided a smaller, 109 sample representative dataset. Of these, we were able to find 94 which were in Genbank, and thus were available, with metadata, in our database. We ran our genotyping pipeline on these sequences, and found 18 clusters, as opposed to the 19 clades found in the nomenclature study. The phylogenetic tree, colored by cluster membership, is shown in Figure 3. To compare the results of our genotyping pipeline with the expert-defined genotypes, we used the Adjusted Rand Index [48], which has an expected value of zero, and a maximum value of 1. The Adjusted Rand Index for this experiment was 0.833, indicating a strong agreement between our results and the clades defined by the WHO/FAO/OIE.

We also performed a detailed examination of the trees produced by the CCV method with those from the study. We found that members of Clade 2.3.1 were found in two distinct groups on our tree, one of which was quite distant from the rest of the samples in the tree. Upon looking at the sequences, we found that these were much shorter (~1000 bp) than the rest of the sequences (~1600 bp), indicating that these sequences were most likely HA1 sequences, rather than the full HA coding sequence, even though they were labelled full HA. This highlights a problem with the quality of data that currently exists in the databases. These inconsistencies in the data can significantly distort the results of the various sequence analysis methods.

### Bacterial Genomes

We investigated is our implementation can be used for larger and more complex genomes. We acquired 27 full length *Actinomycetes *genomes from the Tuberculosis Database at the Broad Institute and ran them through our pipeline. We were able to produce trees that had the same topology as those generated by the Broad in about 30 minutes compared to several hours for them using traditional tools (Brian Weiner, personal communication, from unpublished data). In addition, we compared the trees produced using the concatenation of the predicted proteins of the same set of genomes and we got similar results in both time to produce and the topology of the trees. However, it should be noted that the length of the branches are different.

### MapReduce

During the development of the CCV code, we ran into memory bottle necks that required extensive coding to minimize. In addition, it was noted that several of the steps could be parallelized. We examined Hadoop to determine if we could utilize it for parallelizing our code across commodity hardware in a fault-tolerant way. We were able to code most of our algorithms using Hadoop. The few that we did not code were the neighbour-joining tree and the affinity propagation clustering algorithms. We provide a modified version of Panjo [47], a neighbour-joining algorithm, that uses the output of our Hadoop cosine distance matrix, which is in row major (packed) order. We also can output the matrix in the Phylip square format. We were able to generate a neighbour-joined tree for 10,270 16S samples in 106 minutes using our Rocks Cluster of 30 machines, which we were not able to compute at all using our code on a single machine.

## Conclusions

**Figure 4:**
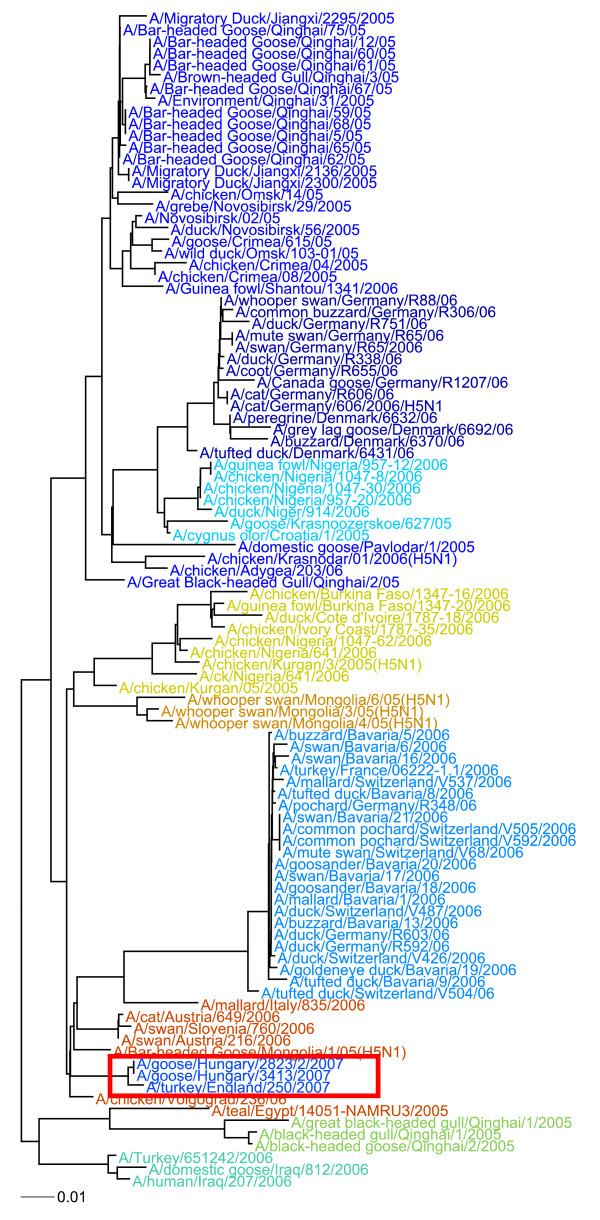
**Integration of Metadata With Genotyping Results**. Clustering of HA genes from the 2007 United Kingdom H5N1 outbreak with other samples isolated around the same time. This shows how researchers can combine genotyping with the metadata. Colors on the map represent the cluster membership. The red box indicates the cluster containing the UK Turkey Sample, along with the two Hungarian samples. Note the dates for the UK and Hungary samples (Light Blue) -- these dates were only provided at the year level in Genbank, even though more accurate dates can be inferred from other sources.

We have described a fast and accurate method for computational genotyping, using both human and avian *Influenza *as a model organism, full length *Actinomycetes *genomes, and 13 S samples. This method utilizes techniques that are faster than traditional methods for both sequence comparison and clustering. Our method produces genotypes that closely match those produced by expert analysis. In addition to providing discrete genotypes with minimal human intervention, the complete composition vector based method produces trees that correlate highly with those produced by sequence alignment and maximum likelihood methods, giving scientists a visualization of the data that they are familiar with in a fraction of the time. Possible uses of these tools include displaying the genotypes and associated metadata on a timeline or map, to show the geospatial and temporal distribution of the pathogen population (Figure 4). Finally, our MapReduce implementation should handle tens of thousands of bacterial size genomes and genomes of complex Eukaryote organisms (we have tested this with several *Fusarium sp*. and got similar trees, data not shown), such as those being produced from current and next generation sequencers, providing a method to analyze these large datasets.

## Availability

Project name: Nephele

Project home page: http://code.google.com/p/nephele/

Operating system: Linux, Mac OS X, Unix

Programming language: Java and C

License: Apache License 2.0

## Competing interests

The authors declare that they have no competing interests.

## Authors' contributions

MEC, MP, and SM wrote source code for CCV. MEC wrote source code for Hadoop CCV. MEC, LH, and MP conceived of the study, and participated in its design and coordination and helped to draft the manuscript. All authors have read and approved the final manuscript.

## Supplementary Material

Additional file 1 **Representative Standard Nomenclature Dataset of H5N1 Genotypes**. A set of 94 sequences representing WHO expert-defined genotypes (http://www.who.int/csr/disease/avian_influenza/guidelines/nomenclature/). Click here for file

Additional file 2 **Clustering of Eight Genes from Influenza H3N2 Viruses (HA, M1, NA, NP, NS1, PA, PB1, PB2)**. This dataset consists of 155 samples, taken from New York State during the 1999-2000, 2001-2002, 2002-2003, and 2003-2004 flu seasons. Click here for file
