# Supplementary figures and images for: Nephele: genotyping via complete composition vectors and MapReduce
Source: Source Code Biol Med. 2011 Aug 18;6:13. doi: 10.1186/1751-0473-6-13 (PMC3182884; doi:10.1186/1751-0473-6-13)

HA

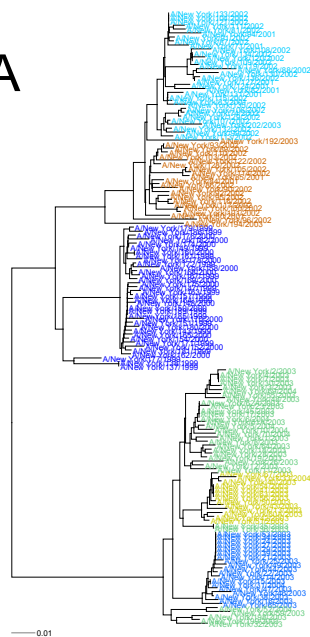

M1

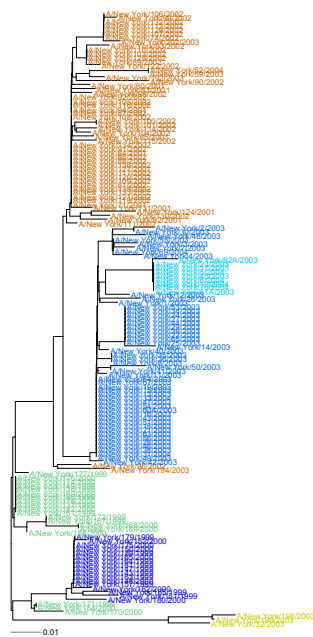

NA

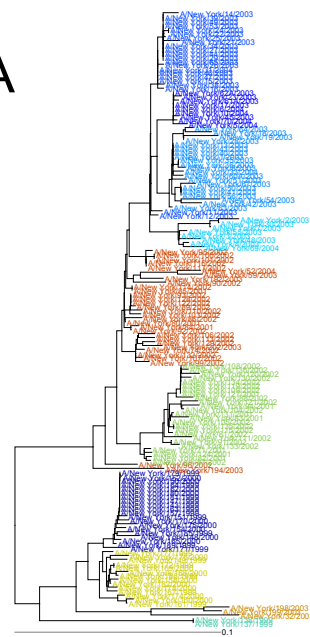

NP

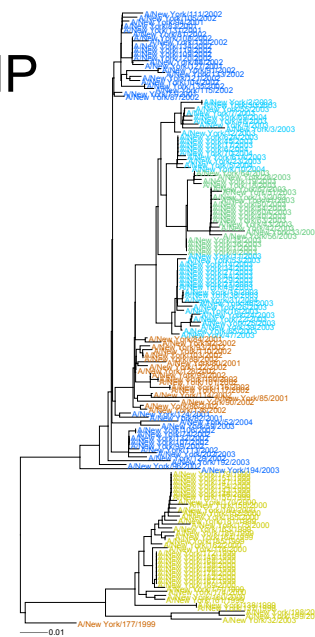

NS1

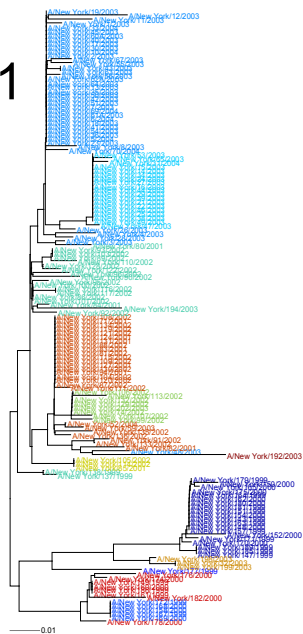

PA

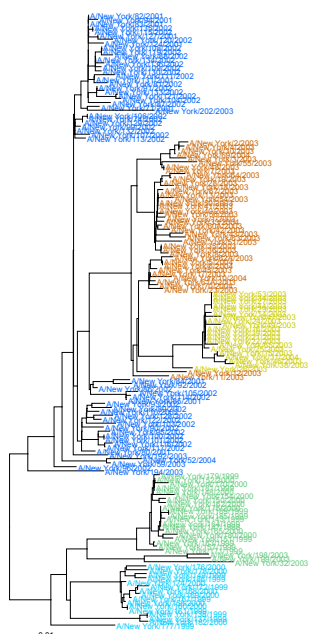

PB1

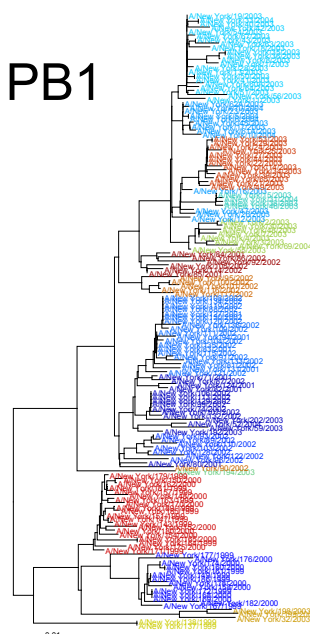

PB2

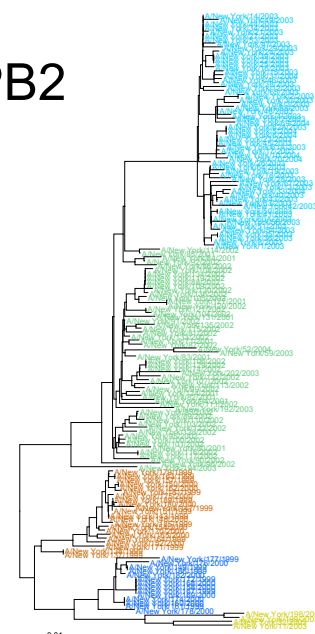

Supplement: Additional file 2 — Clustering of Eight Genes from Influenza H3N2 Viruses (HA, M1, NA, NP, NS1, PA, PB1, PB2). This dataset consists of 155 samples, taken from New York State during the 1999-2000, 2001-2002, 2002-2003, and 2003-2004 flu seasons. [file 1751-0473-6-13-S2.PDF]
